# A Cell Component-Related Prognostic Signature for Head and Neck Squamous Cell Carcinoma Based on the Tumor Microenvironment

**DOI:** 10.1155/2022/6022869

**Published:** 2022-06-25

**Authors:** Siyu Li, Yajun Gu, Junguo Wang, Dengbin Ma, Xiaoyun Qian, Xia Gao

**Affiliations:** ^1^Department of Otolaryngology Head and Neck Surgery, Affiliated Drum Tower Hospital, Medical School of Nanjing University, Jiangsu Provincial Key Medical Discipline (Laboratory), No. 321 Zhongshan Road, Nanjing 210008, China; ^2^Research Institute of Otolaryngology, No. 321 Zhongshan Road, Nanjing 210008, China

## Abstract

Head and neck squamous cell carcinoma (HNSCC) is a heterogeneous disease with a high mortality rate. The tumor microenvironment (TME) is composed of numerous noncancerous cells that contribute to tumorigenesis and prediction of therapeutic effects. In this study, we aimed to develop a cell component-related prognostic model based on TME. We screened cell component enrichments from samples in The Cancer Genome Atlas (TCGA) HNSCC cohort using the xCell algorithm. Univariate Cox and multivariate Cox regression analyses were performed to establish an optimal independent risk model. The prognostic value of the model was further validated using Gene Expression Omnibus datasets. We found that patients in the low-risk group had a better outcome and activated immunity and may benefit more from the immune checkpoint inhibitor therapy. We also explored microRNAs (miRNAs) that may regulate these identified cell components, and 11 miRNA expression levels influenced the overall survival time. Moreover, their target mRNAs were differentially expressed in TCGA cohort and enriched in pathways of cell cycle pathways, extracellular matrix receptor interaction, human papillomavirus infection, and cancer. In summary, our cell component-related signature was a promising prognostic biomarker that provides new insights into the predictive value of nontumor components in the TME.

## 1. Introduction

Head and neck squamous cell carcinoma (HNSCC) is the sixth most prevalent malignancy globally, with a high mortality rate of 40–50% [1], comprising a heterogeneous group of tumors originating from the mucosal epithelium in the oral cavity, oropharynx, larynx, and hypopharynx. Notably, approximately 60% of HNSCC-induced mortality is caused by a high rate of local recurrence [2]. In general, tobacco use and alcohol consumption are the most important risk factors for HNSCC, and as an emerging risk factor, infection with oncogenic strains of human papillomavirus (HPV) has been proven to be closely connected with oropharyngeal cancers (>70%). Surgery alone or combined with the following radiation or chemoradiotherapy is the current standard treatment for HNSCC, depending on the tumor origin and clinical stage [3].

In recent years, the concept of the tumor microenvironment (TME) has improved the understanding of tumors. TME comprises cancer and noncancerous cells, including fibroblasts, endothelial cells, neurons, adipocytes, and adaptive and innate immune cells, as well as noncellular components. Reciprocal interactions between cancer cells and noncancerous cells in the TME contribute to the evolutionary development of cancer [4–6]. In HNSCC tissues, in addition to the cancer cells, the TME consists of numerous noncancerous cell types, including immune and stromal cells, that can interact with malignant cells. Numerous studies have shown that immune cell infiltration in HNSCC plays a vital role in determining the development and prognosis of HNSCC [7]. Although several clinical trials have verified that HNSCC responds well to immunotherapy agents [8], including epidermal growth factor receptor monoclonal antibody and anti-programmed cell death 1 (anti-PD1), only a subset of patients can benefit from these agents [9]. This indicates that focusing solely on the immune cells in the TME is insufficient. Hence, in this study, we explored the specific cell component enrichments in HNSCC using xCell, which provided a landscape of 64 cell components, including immune, stromal, and other cell types. Based on this, we established a prognostic risk model using univariate and multivariate Cox regression analyses, and we identified subgroups of patients with low and high prognostic risks. The high-risk group showed a significantly higher copy number variation (CNV), aneuploidy score, and tumor purity, with lower response sensitivity to the immune checkpoint inhibitors (ICIs) than the low-risk group. Given the important role of microRNAs (miRNAs) in cell regulation and communication, we obtained a miRNA map related to altered cell components in HNSCC by gene set enrichment analysis (GSEA). These results provide new insights into the potential prognostic and therapeutic targets in HNSCC.

## 2. Materials and Methods

### 2.1. Data Acquisition and Processing

For the training set, the tumor mRNA and miRNA profiles (Fragments Per Kilobase Million (FPKM) values) were downloaded from The Cancer Genome Atlas (TCGA) database (https://portal.gdc.cancer.gov) (tumor samples, *n* = 500; normal control samples, *n* = 44). In addition, the corresponding clinical information on tumor samples, including age, sex, TNM stage, histologic grade, HPV status, overall survival (OS) time, and status, was obtained (*n* = 500, excluding samples with incomplete survival data). Two independent datasets were extracted from the Gene Expression Omnibus (GEO) repository (https://www.ncbi.nlm.nih.gov/geo/) for subsequent validation. GSE65858 included 270 HNSCC samples, and GSE41613 included 97 patients.

### 2.2. Cell-Type Enrichment Analysis

The xCell algorithm is a novel gene signature-based strategy used for cell composition analysis that can distinguish between 64 cell types. The relative enrichment score (ES) of cell components in heterogeneous samples was determined using the xCell algorithm with transcriptome data.

### 2.3. Establishment and Validation of a Prognostic Predictive Signature

Uni- and multivariable Cox regression models were built using the Coxph function in the “survival” package. Univariate analyses were performed for OS time and status along with cell component enrichment in the TME analyzed by xCell. The criterion of *p* < 0.05 was selected as the filtering threshold. Next, we used multi-Cox regression analysis to build a prediction model; we selected nonzero regression coefficients to identify the optimal cell component sets and calculated the risk coefficient (Coef) of the cell component. The risk value for eachpatient was obtained using the following formula: risk score = (ES of cell component 1∗Coef 1) + (ES of cell component 2∗Coef 2) + (ES of cell component 3∗Coef 3) + ⋯+(ES of cell component *n*∗Coef *n*). According to the above formula, the median value of the risk score was used as the truncated cut-off value for high- and low-risk group demarcations. The same risk formula was used to compute the risk scores for patients in the GEO datasets. Curves for OS were estimated by the Kaplan–Meier method, and the survival differences between subgroups were compared with the log-rank test. In addition, time-dependent receiver operating characteristic (ROC) curves and area under the curve (AUC) at 1 and 3 years were calculated to evaluate the prognostic ability of the above model. Kaplan–Meier curves and ROC curves were generated by the “survival” package in the R software. Independent datasets from the GEO repository were used for further validation.

### 2.4. CNV Estimation, Aneuploidy Score, and Tumor Purity Estimation

The CNV score, aneuploidy score, and tumor purity data were obtained from the Supplementary files of “The Immune Landscape of Cancer” [10].

### 2.5. Identification of Differentially Expressed Immune Checkpoint Genes between the Low- and High-Risk Groups

The differentially expressed immune checkpoint genes in TCGA-HNSCC were identified with limma package in the R software. Genes with an absolute log2 − fold change (FC) > 1 and an adjusted *p* value < 0.05 were considered for further analysis.

### 2.6. Identification of miRNAs Related to Cell Components

To filter miRNAs related to specific cell components in the prognostic model, we calculated the correlation of mRNAs with specific miRNAs by Pearson correlation analysis and ranked mRNA based on the rank score (RS) as follows:

(1) $$\begin{array}{c} {\text{RS}}_{ij}=-\log10\left(p_{ij}\right)*r_{ij}, \end{array}$$

where the p_ij_ and r_ij_ represent the *p* value and correlation index of miRNA *I* and gene *j*, respectively.

Next, we replaced the genes in GSEA pathways with marker genes of cell components in the prognostic model to detect miRNAs related to specific cell components. Cytoscape was used to visualize the interaction network between the miRNAs and target cell components.

### 2.7. Enrichment Analysis of miRNA-Targeted Genes

MiRNA target genes were predicted using an online miRNA target prediction database (http://mirdb.org). The limma package was used to analyze differentially expressed genes (DEGs) between the HNSCC and control groups in TCGA cohort, and an adjusted *p* value < 0.05 and |logFC| > 1 were set as the selection thresholds of DEGs. We ranked these miRNA-targeted genes included in DEGs by the value of logFC. GSEA, Gene Ontology (GO), and KEGG enrichment analyses were conducted by the clusterProfiler package (version 3.12.0) and enrichplot package (version 1.4.0).

### 2.8. Statistical Analyses

All statistical analyses were performed using R software (version 3.6.1). Gene expression data were transformed for log2 in all analyses. The Wilcoxon test was used to compare two groups, while the Kruskal-Wallis test was used to compare multiple groups. The chi-square test was used to analyze the relationship between bicategorical variables. The Kaplan–Meier plot was employed to generate survival curves for subgroups. The relationship between cell components was analyzed by Spearman rank correlation analysis based on signature classifiers and pathways, and an index value of more than 0.5 was considered statistically significant.

## 3. Results

### 3.1. Overall Protocol and Dataset Acquisition

**Figure 1:**
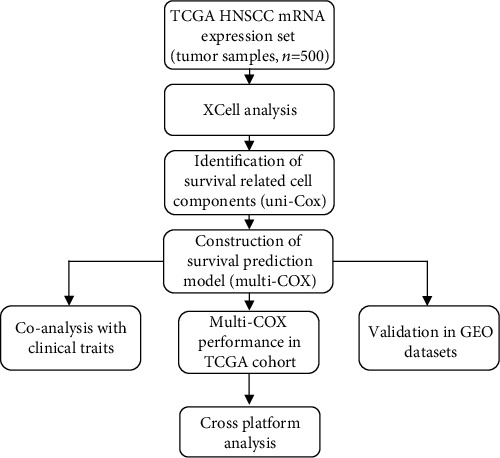
Workflow of the study.

The workflow of this study is illustrated in Figure 1. TCGA-HNSCC samples (*n* = 500) and GEO datasets (GSE65858, *n* = 270; GSE41613, *n* = 97) were enrolled in the current study (excluding samples with incomplete clinical data). Patients in TCGA dataset were diagnosed with primary HNSCC from 1992 to 2013; the primary tumor sites included the oral cavity (321 cases), oropharynx (40 cases), larynx (111 cases), hypopharynx (10 cases), and alveolar ridge (18 cases). Patients in GSE65858 were diagnosed with primary or metachronous secondary HNSCC at the University Hospital Leipzig, and those with a prior history of other cancers were excluded. Of these, 106 cases originated from the oropharynx, 48 from the larynx, 33 from the hypopharynx, and 83 from the oral cavity [11]. GSE41613 contained 97 cases of HPV-negative OSCC at the Fred Hutchinson Cancer Research Center between 2003 and 2007 [12]. The corresponding clinical data included age, sex, clinical stage, and TNM stage (Table S1).

### 3.2. Establishment of a Prognostic Risk Model for HNSCC

**Figure 2:**
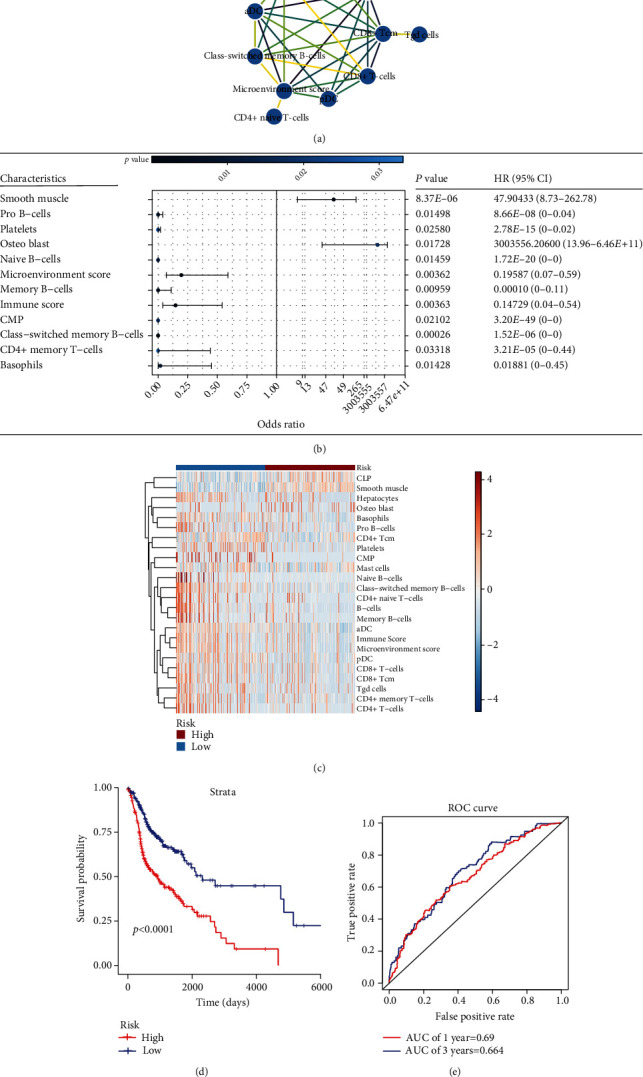
Establishment of a prognostic risk model for HNSCC. (a) Interaction network of highly related cell components. Analyzed by Spearman correlation analysis; |correlation index| > 0.5; as edge color from yellow to purple, the correlation index increases. (b) Forest plot of 12 prognostic components. HR: hazard ratio; CI: confidence interval. (c) Heatmap showing differential expression of 24 cell components that related to overall survival between the low- and high-risk group. (d) Kaplan–Meier plots of the prognostic model, with red line representing the high-risk group and blue line representing the low-risk group. (e) Receiver operating characteristic (ROC) curves for the low- and high-risk groups, with area under the curve (AUC) scores.

The transcriptome profile from TCGA database was used as a training dataset, and the cell component enrichments in HNSCC were presented by the xCell algorithm. Univariate Cox regression analysis was used to investigate the types of TME cells that were associated with HNSCC prognosis, and 24 prognostic cell components related to OS were identified (*p* < 0.05). Of these, 21 were protective (hazard ratio (HR) < 1.0) and the other three were risky (HR > 1.0) (Table S2). The interaction network showed that T lymphocytes and B lymphocytes are highly correlated in HNSCC based on the correlation index (|correlation index| > 0.5, Figure 2(a)), which indicates that adaptive immune responses play an important role in the development of HNSCC. Subsequent multivariate analysis provided a combination of 12 eligible prognostic components. The forest plot showed the HR with a 95% confidence interval (CI) and *p* value of these 12 components (Figure 2(b)). Higher enrichments of pro-B cells, platelets, naive B cells, microenvironment score, memory B cells, immune score, common myeloid progenitor (CMP), class-switched memory B cells, CD4+ central memory cells (CD4+ Tcm), and basophils tended to predict better survival, whereas higher expressions of smooth muscle and osteoblasts tended to predict a poorer survival. The risk score representing a new prognostic signature of each patient with HNSCC was calculated using the following formula: risk score = [(−4.31) × ES_Basophils_] + [(−7.48) × ES_CD4+Tcm_] + [(−14.00) × ES_Class−switched memory B cells_] + [(−81.26) × ES_CMP_] + [19.05 × ES_Memory B cells_] + [(−68.36) × ES_naive B cells_] + [18.95 × ES_Osteoblast_] + [(−45.91) × ES_Platelets_] + [(−18.19) × ES_pro−B cells_] + [2.51 × ES_Smooth muscle_] + [5.71 × ES_ImmuneScore_] + [(−3.94) × ES_MicroenvironmentScore_]. Based on the risk score, we redivided the HNSCC patients into the high-risk (*n* = 250) and low-risk groups (*n* = 250) according to the median risk score (median risk score = 1.0837). The heatmap showed that patients in the high-risk group had significantly less immune-related cell enrichment and higher smooth muscle cell enrichment (Figure 2(c)). Patients in the high-risk group had substantially worse outcomes than those in the low-risk group (Figure 2(d)). To further determine the robustness of the signature in predicting the prognosis of HNSCC, ROC analysis was performed. The ROC curves showed a 1-year survival AUC value of 0.69 and a 3-year survival AUC value of 0.664, which indicated moderately good predictive power (Figure 2(e)).

### 3.3. Validation of the Established Prognostic Risk Model

**Figure 3:**
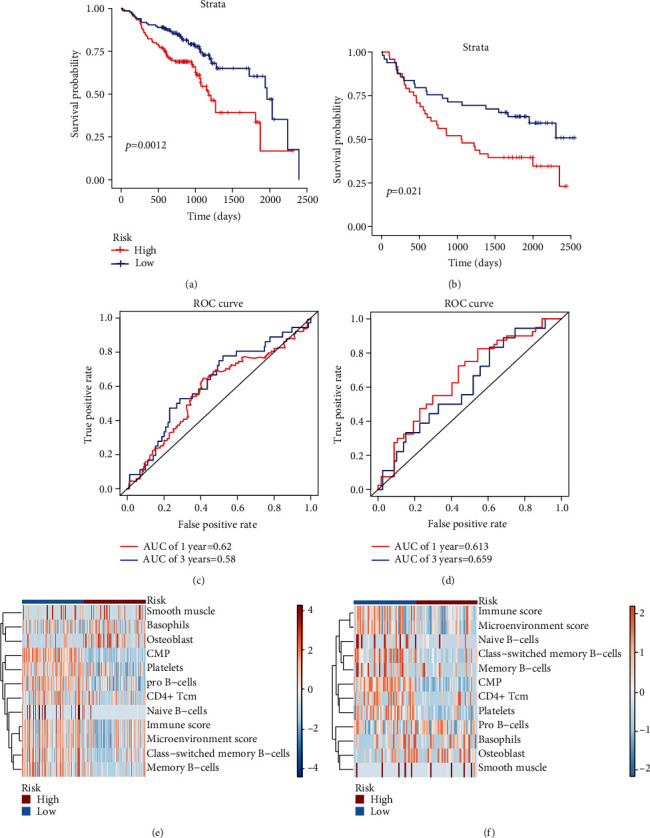
Validation of the established prognostic risk model. (a, b) Kaplan–Meier plots of prognostic model performance in GSE65858 and GSE41613, with red line representing the high-risk group and blue line representing the low-risk group. (c, d) Receiver operating characteristic (ROC) curves for the low- and high-risk groups in GSE65858 and GSE41613, with area under the curve (AUC) scores. (e, f) Heatmap showing differential expression of 12 elements in the prognostic model between the low- and high-risk groups in GSE65858 and GSE41613.

The GSE65858 and GSE41613 datasets were used as external validation datasets to test the prognostic risk model. Patients in the high-risk groups had worse OS than those in the low-risk groups (Figures 3(a) and 3(b)). The time-dependent ROCs of GSE65858 and GSE41613 showed a predictive power similar to those of TCGA-HNSCC dataset (Figures 3(c) and 3(d)). The enrichment profiling of the 12 cellular components is presented in heatmaps (Figures 3(e) and 3(f)).

### 3.4. Prognosis Risk Score Was an Independent Risk Factor for HNSCC

**Figure 4:**
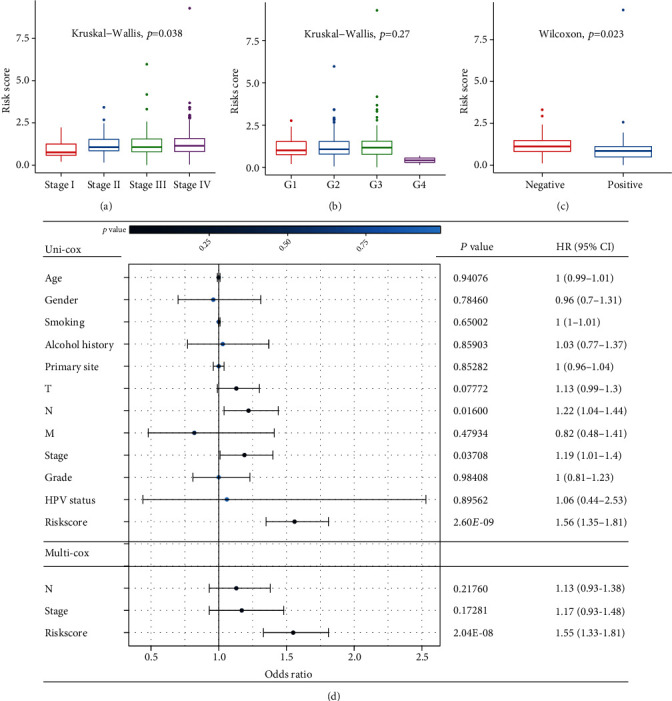
Coanalysis with clinical traits. (a-c) Risk score in different groups of clinical stages, pathological grades, and HPV status. (d) Forest plot of risk score and clinical traits. HR: hazard ratio; CI: confidence interval. A univariate Cox hazard ratio analysis demonstrated that N stage (*p* < 0.05, HR = 1.22, 95% CI [1.04–1.44]), clinical stage (*p* < 0.05, HR = 1.19, 95% CI [1.01–1.40]), and risk score (*p* < 0.0001, HR = 1.56, 95% CI [1.35–1.81]) were statistically different. Only risk score (*p* < 0.0001, HR = 1.55, 95% CI [1.33–1.81]) presented as an independent prognostic predictor by multivariate Cox regression.

To further assess whether the risk score is an independent risk factor, we explored the differences in traditional risk factors, such as age, sex, smoking, alcohol consumption, TNM stage, pathology grade, and HPV status, between the low- and high-risk groups in TCGA-HNSCC dataset. Late-stage and HPV-negative patients tended to present with higher risk scores (Figures 4(a) and 4(c)). However, there was no difference in the clinical stages and HPV status between the low- and high-risk groups (Figure S1d, e). No significant difference was observed in the risk scores for age, sex, or pathological grade (Figure 4(b); Figure S1a, b), and the low-risk group showed a longer OS time (Figure S1c). Univariate and multivariate Cox regression analyses revealed that the risk score was an independent risk factor for HNSCC (Figure 4(d)).

### 3.5. CNV, Aneuploidy Score, and Tumor Purity Estimation

**Figure 5:**
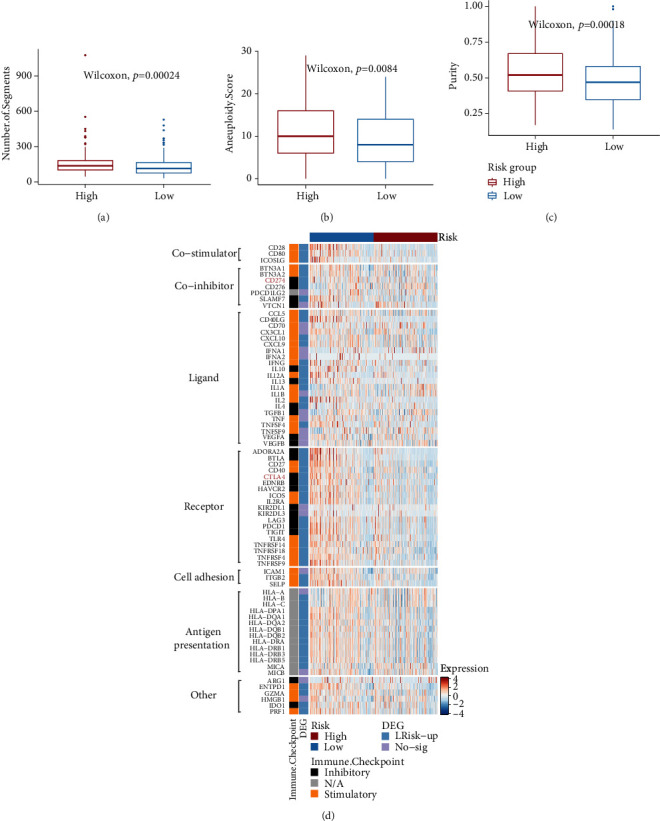
Crossplatform analysis and expression of immune checkpoint genes TCGA cohort. (a–c) Boxplots showed that the low-risk group presented with lower copy number variation (CNV), aneuploidy score, and tumor purity (*p* < 0.001). (d) Heatmap presented with differential expression of immune checkpoint genes in TCGA cohort. Numerous immune checkpoint genes were significantly higher in the low-risk group (log2 − fold change (FC) > 1 and an adjusted *p* value < 0.05, “LRisk-UP” in the annotation on the right means that the expressions of these genes are upregulated in the low-risk group, and “No-Sig” means that there is no statistical difference between these two groups).

To detect the heterogeneity of HNSCC, we investigated the CNV, aneuploidy score, and tumor purity in the high- and low-risk groups. We found that patients in the low-risk group had a lower CNV, aneuploidy score, and tumor purity than those in the high-risk group (Figures 5(a)–5(c)).

### 3.6. Benefit of ICI Therapy in Classifier-Defined Subgroups

ICI therapy has proven to be an effective treatment for patients with recurrent or refractory HNSCC; however, only a portion of patients benefit from this treatment. Hence, identifying patients who can benefit most from this therapy is crucial. To explore the therapeutic effects of ICIs between subgroups, we analyzed the expression of known immune checkpoint genes in TCGA cohort. Overall, the low-risk group had higher immune activation, such as antigen presentation, costimulators, and receptors, than the high-risk group (Figure 5(d)). Among them, CTLA4 and CD274 (PD-L1), two classical targets of ICIs, were also upregulated in the low-risk group, indicating that patients in the low-risk group may benefit more from CTLA4 and PD-L1 inhibitor therapies.

### 3.7. miRNAs Related to Cell Components in the Prognostic Model

**Figure 6:**
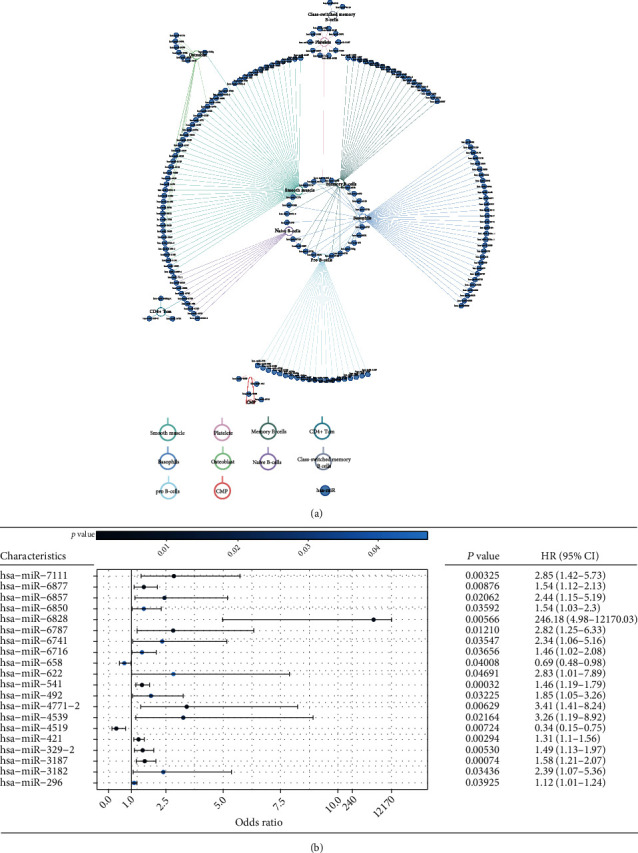
MicroRNAs related to cell components in the prognostic model. (a) Network map showed miRNAs that are related to 10 cell components in the prognostic model. (b) Forest plot of 20 miRNAs related to overall survival time and status. HR: hazard ratio; CI: confidence interval. A univariate Cox hazard ratio analysis demonstrated that among them, 18 miRNAs were risky (HR > 1.0) and 2 miRNAs were protective (HR < 1.0).

**Figure 7:**
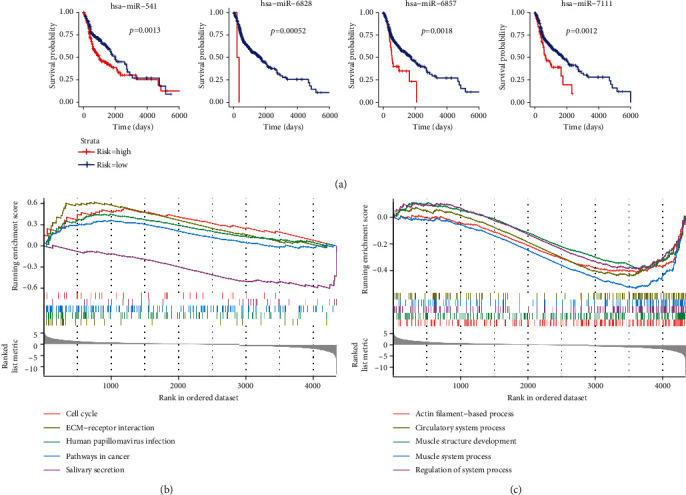
Kaplan–Meier plots of microRNAs related to cell components in the prognostic model and GSEA of their targeted genes. (a) Representative Kaplan–Meier plots of microRNAs related to cell components in the prognostic model, with red line representing the high-risk group and blue line representing the low-risk group. (b) KEGG pathway enrichment of 11 miRNA targeted genes in TCGA-HNSCC cohort. (c) GO enrichment of 11 miRNA targeted genes in TCGA-HNSCC cohort.

miRNAs are a class of single-stranded noncoding RNAs, and their dysregulation is related to the promotion, migration, and invasion of tumor cells. In the TME, miRNAs are involved in the recruitment and modification of stromal cells by tumor cells and mediate interactions between various immune and cancer cells [13, 14]. To determine the underlying mechanism, we obtained a map of miRNAs related to different cell components in the prognostic model. Using a modified GSEA, we identified 173 miRNAs related to 10 cell components in the prognostic signature. Among them, 54 miRNAs were related to smooth muscle; 47, to basophils; 32, to memory B cells; 27, to pro B cells; 14, to osteoblast; 12, to naive B cells; 11, to platelets; 4, to CD4+ Tcm; 4, to CMP; and 3, to class-switched memory B cells (Figure 6(a)). Next, we performed univariate Cox regression analysis on these miRNAs and found that 20 miRNAs were related to tumor progression, including two protective miRNAs (HR < 1.0) and 18 risky miRNAs (HR > 1.0) (Figure 6(b)). By Kaplan–Meier curves, 15 miRNAs were found to be closely correlated with patient outcomes (Figure 7(a); Figure S2). Combining these two results, we found that 11 miRNAs were in common, including hsa-miR-329-2, hsa-miR-421, hsa-miR-4519, hsa-miR-4539, hsa-miR-541, hsa-miR-622, hsa-miR-6741, hsa-miR-6787, hsa-miR-6828, hsa-miR-6857, and hsa-miR-7111. Using an microRNA target prediction database, we obtained the target genes of these 11 miRNAs. Then, we explored the expression changes between the tumor and control samples in TCGA cohort, and mRNAs with an adjusted *p* value < 0.05 were selected for GSEA. GSEA of these target mRNAs showed that cell cycle, extracellular matrix- (ECM-) receptor interaction, HPV infection, and cancer pathways were the top four enriched pathways (Figure 7(b)). Moreover, GO revealed that these genes primarily function in the actin filament-based process, muscle structure development, and muscle system process (Figure 7(c)). This suggests that the 11 miRNAs may regulate the interaction of cell components in HNSCC through these pathways and determine the occurrence and development of HNSCC.

## 4. Discussion

The TME of HNSCC is a mixture of immune cells and stromal cellular elements. This complex and dynamic microenvironment is now recognized to be involved in promoting and inhibiting tumor growth, invasion, and metastasis. At present, single-cell RNA-seq has been widely used to explore TME cell interactions in various types of tumors. However, there were few human HNSCC single-cell RNA-seq profiles (GSE164690, GSE103322, and GSE150321). Unfortunately, the patients included in these studies were restricted by single origin and HPV status, which may have limited our study. Hence, we used xCell, a cell-type enrichment analysis based on bulk gene expression data, to explore the cell component changes in HNSCC. The prognostic model contained 10 different cell components of HNSCC, most of which were immune cells. Accumulating evidence has shown that the outcome of HNSCC depends on the balance between antitumor and immunosuppressive immune cells, and the disruption of this balance always promotes tumor progression and influences survival time [15]. Immune components account for a large part of our signature, including tumor-infiltrating lymphocytes (pro-B cells, naive B cells, memory B cells, class-switched memory B cells, and CD4+ Tcm cells), myeloid lineage cells (basophils), and stem cells (platelets and CMP). The high enrichment of these immune components, immune score, and microenvironment score correlated with an improved OS time, which is in accordance with previous studies that patients with an active immune class had a significantly favorable prognosis. B lymphocytes are important cells that participate in the immune response to cancer. B cells do not only produce antibodies against a tumor but also serve as antigen-presenting cells to activate T cells. Moreover, B cells can also shape the immune response in the TME toward a pro- or antitumor direction by secreting distinct cytokines [16]. In contrast to T cells, B cells have not been well described in HNSCC. CD20+ B cells have been reported to influence the prognosis of various tumors, including non-small-cell lung cancer, gastric cancer, melanoma, and colorectal cancer. Lechner et al. found that memory B cells, which play key roles in antibody-mediated responses, account for the largest proportion of B cells in the TME of HNSCC and were speculated to have antitumor activity [17]. Increased peritumoral B cells in lymph node metastases have been proven to be associated with improved outcomes of HNSCC [18]. High expression of CD19 and IGJ, two surface markers of B cells, results in dramatically improved 3-year overall survival, and the depletion of B cells can promote tumor growth [19]. Similar to these results, in this study, the enrichments of pro-B cells, naive B cells, memory B cells, and class-switched memory B cells were relatively higher in the low-risk group, with improved OS and antitumor immunity. For T cells, our analysis indicated that CD4+ Tcm correlated with a good outcome in patients with HNSCC, and this is consistent with previous studies [20]. Platelets can mediate the TME by secreting granules. The cargo from these granules is released into the extracellular environment, leading to platelet aggregation and vasoconstriction. It then regulates the cell proliferation through the secretion of numerous growth factors [21]. Our study revealed that the enrichment of platelets improved the patient outcomes from the perspective of transcriptome information.

As HNSCC tumors progress, stromal cells have been shown to play direct and indirect roles in facilitating HNSCC invasion [22]. Tumor cells can coopt reactive stromal cells and convert them into tumor-associated stromal cells, which promote extracellular matrix remodeling, cell migration, drug resistance, and immunosurveillance evasion through the production of a variety of growth factors, chemokines, and cytokines [23]. We found that enrichment in stromal cells, including smooth muscle cells and osteoblasts, contributed to poor patient outcomes. Evidence suggests that osteoclast activation and subsequent bone destruction are involved in the pathological process of HNSCC, which supports our analysis [24]. Although there is a consensus that stromal *α*-SMA^+^ myofibroblasts can play an important role in creating a permissive environment for tumor invasion in oral and laryngeal squamous cell carcinoma [25], there is still a lack of understanding of smooth muscle cells in the TME, thus requiring further validation and research.

It is well established that the tumor cell coevolves with the surrounding microenvironment, and during tumor progression, there is substantial crosstalk between various cell types in the TME, which promotes tumor growth and development. miRNAs are important molecules involved in posttranscriptional regulation. Studies have shown that miRNAs play an important role in mediating and controlling various cell interactions in the TME and may serve as prognostic indicators in many types of cancers, including HNSCC [13, 14, 26, 27]. We identified 173 miRNAs involved in the regulation and communication of cell components in the prognostic signature, and 11 miRNAs were confirmed to influence the OS of patients with HNSCC. Previous studies have demonstrated that miR-421 upregulation is associated with the occurrence of gastric, liver, and lung cancer [28–30]. Moreover, knockdown of miR-421 remarkably suppresses the proliferation, migration, and invasion of non-small-cell lung cancer cells [31]. Ji et al. illustrated that miR-421 promotes cell invasion and proliferation in HNSCC cell lines [32], which is in accordance with our results. Studies have shown that miR-541 inhibits the proliferation, migration, and invasion of osteosarcoma cells and that miR-622 downregulation is significantly associated with poor prognosis in various other tumors; these are contrary to our predicted miRNA functions in HNSCC [33–35]. However, there have been few studies on their functions in HNSCC. The miRNAs miR-329-2, miR-4519, miR-4539, miR-6741, miR-6787, miR-6828, miR-6857, and miR-7111 have not been reported in previous tumor-related studies. Thus, additional research is warranted to determine their functions in patients with HNSCC. Deregulated cell proliferation, in combination with suppressed cell death, is an intrinsic factor in tumor occurrence and progression. KEGG enrichment analysis showed that these miRNA-targeted genes were primarily enriched in cell cycle, ECM receptor interaction, HPV infection, and cancer pathways, which all play important roles in tumor. Mounting evidence supports that the idea that cell cycle in tumor cells is coupled with immune behavior and miRNAs can affect the cell cycle by inhibiting or initiating cell division [36–38]. Among these miRNAs, miR-421 and miR-622 were reported to be involved in tumor progressing via cell cycle dysregulation previously [30, 39], while others have not been studied. The ECM is an important noncellular component of the TME, and alterations in its density and composition contribute to tumor growth and progression [40]. Expression of miRNAs is critical for extracellular matrix remodeling, thus regulating cancer cells to initiate cell proliferation, migration, adhesion, and invasion [41]. Our data supported these ideas and gave a clue that the cell components in our model may regulate cancerous cells through secretion of miRNAs, by which to determine the degree of malignancy and the response to treatment in HNSCC. The results also suggest that the 11 miRNAs may participate in these pathways to regulate the interaction of cell components, and inhibition of these miRNAs might provide novel therapeutic opportunities. The GO results revealed that these miRNA-targeted genes mainly function in muscle development, which may support the risk factor, smooth muscle, in our prognostic model. Further research is needed to validate this hypothesis.

ICIs have led to breakthroughs in cancer treatment. To date, the U.S. Food and Drug Administration (FDA) has approved many immune checkpoint drugs, including anti-PD1 and anti-CTLA4, for the treatment of HNSCC. However, a major limitation is the low response rate of patients to ICI therapy. In the present study, we clarified the benefits of ICI therapy in our classifier-defined HNSCC subgroups. Significant differences were detected in immune checkpoint expression between the low- and high-risk groups. Immune checkpoints, including costimulators, coinhibitors, ligands, receptors, and molecules functioning in cell adhesion and antigen presentation, were observed, and most of them were significantly upregulated in the low-risk group, suggesting that the low-risk group may have a better response to immunotherapy. Moreover, the expression of CTLA4 and PD-L1 was upregulated in the low-risk group, indicating that immune inhibition remained in the TME and that ICI therapies are still promising with clinical benefits. Chemokines and their receptors play an essential role in the crosstalk between cells in the TME to promote cancer cell growth and metastasis. Manipulation of chemokine-receptor signaling helps to reshape immune status and modulate ICI responsiveness. Among these, the chemokine receptor 4 (CXCR4) and its ligand, CXCL12, are the most studied in the TME [40]. While no significant difference in the expression of CXCL12 was observed between subgroups in our classifier, this could be attributed to the fact that increased CXCR4/CXCL12 axis activity was found to be associated with metastasis and recurrence of HNSCC [42]; however, our model is not based on this. In contrast, CCL5, CXCL9, and CCL10 were upregulated in the low-risk group; high expression levels of CXCL9 and CXCL10 were correlated with improved OS in most tumors, and anti-PD1 therapy was not beneficial in CXCR3^−/−^ (receptor of CXCL9 and CXCL10) tumor-bearing mice [43]. In addition, many cytokines and CD28 superfamily members were all upregulated in the low-risk group, suggesting that the low-risk group in our model benefited more from ICIs and may have a better outcome.

Our study was based on bulk gene expression data. Although xCell provided a relatively detailed cell component landscape, more precise studies are also needed to validate our model by using single-cell RNA-seq data and clinical practices. In addition to cell components, non-cell components are involved in the TME, and they act as a medium for cell interactions and regulate cell development. The KEGG results of miRNA-targeted genes illustrated a critical role of the ECM in tumorigenesis and progression. Hence, to build a more comprehensive and stable understanding of HNSCC-TME, further research is warranted to characterize the specific mechanisms of both cell and non-cell components in tumorigenesis.

## 5. Conclusion

From lumps to cells, our understanding of tumors and treatment methods is constantly being updated as technology advances. Our cell component-related signature is a promising biomarker for predicting patient outcomes, and it might be a potential prognostic indicator for immune checkpoint therapy response. Further studies are required to validate this prognostic model.

## Data Availability

The datasets analyzed during the current study are available in The Cancer Genome Atlas (TCGA) database (https://portal.gdc.cancer.gov) and the Gene Expression Omnibus (GEO) repository (https://www.ncbi.nlm.nih.gov/geo/).

## Supplementary Materials

Supplementary Materials Supplementary Figure 1: coanalysis with clinical traits. (a) Risk score in different genders. (b) Age distribution in low and high-risk group. (c) Overall survival time in the low- and high-risk group (Wilcoxon, *p* < 0.0001). (d, e) Composition of clinical stages and HPV status in the low and high-risk group. Supplementary Figure 2: Kaplan-Meier plots of microRNAs related to cell components in prognostic model. Supplementary Table 1: clinical characteristics of TCGA-HNSCC and GEO datasets. Supplementary Table 2: information of 24 prognostic cell components related to overall survival by univariate cox regression analysis. HR: hazard ratio; CI: confidence interval.
