# Supplementary material for: A Cell Component-Related Prognostic Signature for Head and Neck Squamous Cell Carcinoma Based on the Tumor Microenvironment
Source: Int J Genomics. 2022 Jun 25;2022:6022869. doi: 10.1155/2022/6022869 (PMC9252828; doi:10.1155/2022/6022869)
Supplement: Supplementary Materials — Supplementary Figure 1: coanalysis with clinical traits. (a) Risk score in different genders. (b) Age distribution in low and high-risk group. (c) Overall survival time in the low- and high-risk group (Wilcoxon, p < 0.0001). (d, e) Composition of clinical stages and HPV status in the low and high-risk group. Supplementary Figure 2: Kaplan-Meier plots of microRNAs related to cell components in prognostic model. Supplementary Table 1: clinical characteristics of TCGA-HNSCC and GEO datasets. Supplementary Table 2: information of 24 prognostic cell components related to overall survival by univariate cox regression analysis. HR: hazard ratio; CI: confidence interval. [file 6022869.f1.docx]

Supplementary Table 1 Clinical characteristics of TCGA-HNSCC and GEO datasets

| Characteristics | | TCGA-HNSCC dataset (n=500) | GSE65858 (n=270) | GSE41613 (n=97) |
| --- | --- | --- | --- | --- |
| Age(years) | <60 | 220 | 153 | 50 |
|  | ≥60 | 280 | 117 | 47 |
| Gender | Female | 134 | 47 | 31 |
|  | Male | 366 | 223 | 66 |
| Survival status | Living | 281 | 176 | 46 |
|  | Dead | 219 | 94 | 51 |
| Pathologic_T | T1 | 45 | 35 | / |
|  | T2 | 131 | 80 |  |
|  | T3 | 96 | 58 |  |
|  | T4 | 172 | 97 |  |
| Pathologic_N | N0 | 170 | 94 | / |
|  | N1 | 65 | 32 |  |
|  | N2 | 165 | 132 |  |
|  | N3 | 7 | 12 |  |
| Pathologic_M | M0 | 187 | 270 | / |
|  | M1/MX | 62 | 0 |  |
| Tumor Stage | Stage I | 29 | 18 | 41 |
|  | Stage II | 69 | 37 |  |
|  | Stage III | 78 | 37 | 56 |
|  | Stage IV | 260 | 178 |  |


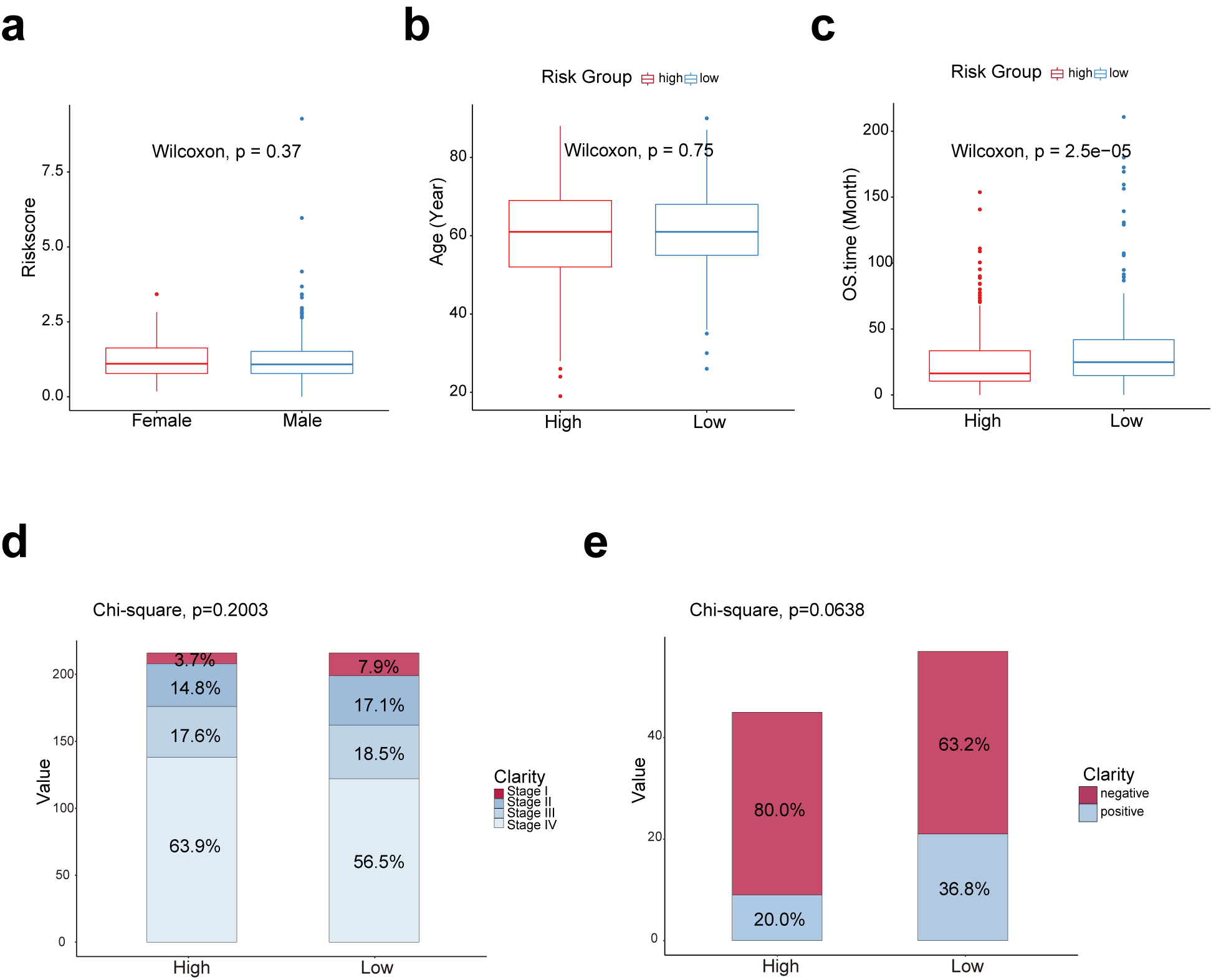


Supplementary Figure 1. Co-analysis with clinical traits.

(a) Risk score in different gender. (b) Age distribution in low and high-risk group. (c) Overall survival time in low and high-risk group (Wilcoxon, p<0.0001). (d-e) Composition of clinical stages and HPV status in low and high-risk group.

Supplementary Table 2. Information of 24 prognostic cell components related to overall survival by univariate cox regression analysis. HR, hazard ratio; CI, confidence interval.

| Cell component | HR (95%CI) | P Value |
| --- | --- | --- |
| aDC | 0.362876849(0.15-0.9) | 0.028423 |
| B-cells | 0.017815196(0-0.2) | 0.001108 |
| Basophils | 0.018811546(0-0.45) | 0.014278 |
| CD4+ memory T-cells | 3.21E-05(0-0.44) | 0.033178 |
| CD4+ naive T-cells | 5.58E-07(0-0) | 0.001232 |
| CD4+ T-cells | 3.59E-11(0-0) | 0.003676 |
| CD4+ Tcm | 0.000431918(0-0.09) | 0.004151 |
| CD8+ T-cells | 0.014762026(0-0.33) | 0.007612 |
| CD8+Tcm | 0.017005507(0-0.28) | 0.00429 |
| Class-switched memory B-cells | 1.52E-06(0-0) | 0.000258 |
| CLP | 95632.08764(219.21-41719780) | 0.000217 |
| CMP | 3.20E-49(0-0) | 0.021022 |
| Hepatocytes | 2.28E-106(0-0) | 0.012747 |
| Mast cells | 1.63E-07(0-0.19) | 0.028418 |
| Memory B-cells | 0.000102715(0-0.11) | 0.009592 |
| naive B-cells | 1.72E-20(0-0) | 0.01459 |
| Osteoblast | 3003556.206(13.96-6.46E+11) | 0.01728 |
| pDC | 0.049130358(0-0.92) | 0.043544 |
| Platelets | 2.78E-15(0-0.02) | 0.025802 |
| pro B-cells | 8.66E-08(0-0.04) | 0.01498 |
| Smooth muscle | 47.90433268(8.73-262.78) | 8.37E-06 |
| Tgd cells | 1.95E-08(0-0) | 0.00453 |
| ImmuneScore | 0.1472938(0.04-0.54) | 0.003635 |
| MicroenvironmentScore | 0.195872975(0.07-0.59) | 0.00362 |

Supplementary Figure 2. Kaplan-Meier plots of microRNAs related to cell components in prognostic model.
